# Ultrasound in medicine from 2014 to 2024: A bibliometric review

**DOI:** 10.1097/MD.0000000000046890

**Published:** 2026-01-09

**Authors:** Zhao Chenhao, Zhou Ruqi, Sun Xiaozhuo, Wang Jiabo, Liu Jiao, Mu Yajuan

**Affiliations:** a College of Traditional Chinese Medicine, Hebei University, Baoding, China; b College of Traditional Chinese Medicine, Capital Medical University, Beijing, China.

**Keywords:** knowledge mapping analysis, medical application, ultrasound, visualization

## Abstract

**Background:**

The unique physical properties of ultrasound technology have led to its increasing recognition in recent years, rendering it an indispensable component in the diagnosis and treatment of various medical conditions. The aim of this study is to investigate the current research status, focal areas, and frontiers of ultrasound technology in the field of medical applications.

**Methods:**

The Web of Science core collection database was interrogated to retrieve relevant data. Biometric analysis employing CiteSpace and VOSviewer software tools was performed to produce comprehensive visualizations depicting authorship patterns, geographic distribution of research contributions, institutional affiliations, journal publications as well as key article themes.

**Results:**

The analysis examined 2459 articles and revealed a substantial increase in the number of publications and citations, indicating a heightened level of research activity and influence. Dietrich CF leads in publications, significantly advancing the field. The research output of China ranks among the global leaders, while Harvard University emerges as the most prolific institution, making substantial contributions to high-quality research, particularly in the domains of artificial intelligence, deep learning, and ultrasound technologies. The emerging fields of ultrasound stimulation and drug delivery are garnering increasing attention and hold the potential to become significant areas of scholarly investigation.

**Conclusion:**

By employing bibliometric methodologies, this study has effectively identified the prominent areas of investigation and dynamic patterns in ultrasound research within the medical field from diverse perspectives. Consequently, it provides an invaluable frame of reference for guiding future research endeavors and facilitating evidence-based scientific decision-making.

## 1. Introduction

The field of ultrasound technology has garnered significant attention in the past decade due to continuous advancements and breakthroughs in science and technology. The medical field has witnessed remarkable progress in ultrasound technology, which now offers indispensable diagnostic and therapeutic capabilities that are noninvasive, convenient, and integral within the healthcare profession.^[1]^

The utilization of ultrasound stimulation has the potential to augment tissue regeneration and offers novel therapeutic possibilities for managing myocardial ischemia through promoting angiogenesis in the ischemic myocardium of rats.^[2]^ The utilization of ultrasound at specific frequencies enables noninvasive and targeted disruption of the blood–brain barrier, thereby augmenting the efficacy of nanodrug release and prolonging mouse survival.^[3]^ The findings of this investigation present potential for the development of innovative approaches to address neurological disorders. The utilization of ultrasound technology has significantly facilitated advancements in neuronal modulation, mouse limb stimulation, and novel applications in rehabilitation medicine.^[4]^ The statement further underscores its efficacy in the treatment of neurological disorders, encompassing Parkinson disease (PD) and depression.^[5,6]^ The integration of ultrasound and nanotechnology has resulted in significant advancements in neural modulation by employing low-intensity ultrasound for precise targeting of specific brain regions, regulation of motor behavior, and alleviation of depressive symptoms. The fragmented nature of the evidence in this field is accountable for the observed lack of rigor in certain research methodologies.

The field of bibliometrics is dedicated to quantifying the volume of scholarly literature, authors, and textual content.^[7]^ The key methodologies employed encompass citation analysis, co-word analysis, and thematic analysis. The examination of disciplinary relationships is facilitated by employing citation analysis, which entails meticulous scrutiny of references in scholarly works. The method of co-word analysis explores thematic associations by quantitatively examining word frequencies in shared documents. Thematic analysis involves systematically categorizing and clustering literary subjects to explore their developmental patterns. This approach is extensively employed for evaluating journal influence, identifying fundamental literature, assessing researcher expertise levels, as well as exploring trends within disciplines and potential avenues for future research.^[8]^ The significance of bibliometrics lies in its capacity to offer empirical data and guidance for scholarly research, thereby fostering advancements across a wide range of disciplines.^[9]^ The advancements in scientometrics, along with the integration of data visualization and knowledge mapping techniques, have facilitated the emergence of sophisticated tools such as CiteSpace (Academic team led by Prof. Chaomei Chen, Drexel University, Philadelphia) and VOSviewer (Leiden University Centre for Science and Technology Studies, Leiden University, Leiden, The Netherlands).^[10,11]^

The current study employs pertinent literature data to investigate the utilization of ultrasound technology in the medical domain. CiteSpace and VOSviewer are utilized for constructing a comprehensive knowledge map and establishing a robust knowledge base. The present investigation provides a comprehensive overview of recent advancements in research, delineates the developmental trajectory, identifies key areas of pioneering research, and predicts future trends within this field.

## 2. Methods

### 2.1. Data collection

**Figure 1.**
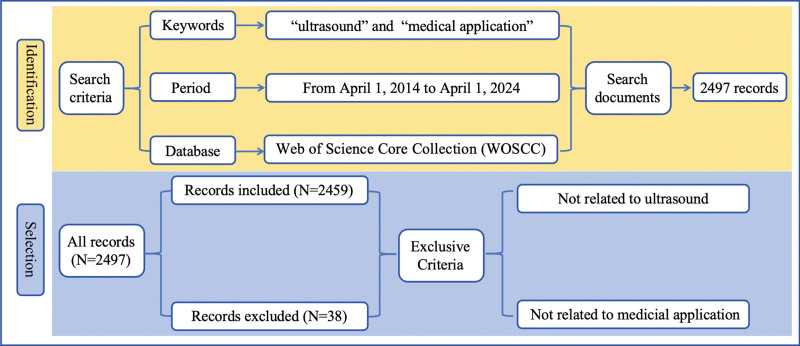
Retrieval methods and inclusion criteria.

The Social Sciences Citation Index and the Science Citation Index Expanded databases are the primary resources employed for conducting comprehensive literature searches within the Web of Science (WOS) platform. The search query formula, as depicted in Figure 1, encompasses the time period spanning from April 1, 2014 to April 1, 2024 and comprises 2 conditions: (TS = ultrasound) and (TS = medical applications). The search yielded a total of 2497 records. Following the exclusion of non-compliant articles, there remained a total of 2459 records. The analysis identified 10 distinct file types within these records. As depicted in Table 1, a total of 1889 records were discovered, accounting for approximately 76.82% of the entire dataset. The dataset consists of a total of 548 article comments, accounting for approximately 22.26% of the overall data records. The remaining 8 categories of literature consist of a total of 30 papers, with 29 articles in early access, 20 retracted publications, 17 editorial materials, 4 book chapters, and only 3 letters and 1 correction in their entirety. Additionally, 1 news article is also included.

**Table 1:** Distribution of document types.

No.	Type of document	TP	SOTC	CA	Proportion/%	*h-index*
1	Article	1889	30,919	27,490	76.82	69
2	Review Article	548	14,595	13,596	22.29	66
3	Proceeding Paper	30	634	628	1.22	15
4	Early Access	29	49	49	1.18	2
5	Retracted Publication	20	120	119	0.81	4
6	Editorial Material	17	211	211	0.69	5
7	Book Chapters	4	84	84	0.16	4
8	Letter	3	17	17	0.12	2
9	Correction	1	0	0	0.04	0
10	News Item	1	0	0	0.04	0

CA = citing articles, SOTC = sum of times cited, TP = total publications.

### 2.2. Data analysis

Through a comprehensive analysis employing VOSviewer and CiteSpace software, a total of 2459 publications were scrutinized to unveil the inherent patterns and developmental trends within the research field. This analytical approach offers invaluable insights for researchers.

The VOSviewer tool is utilized to generate visual maps through the use of coincidence matrices. The analysis process consists of 3 consecutive stages: firstly, the co-occurrence matrix is used to calculate the similarity matrix; secondly, VOS mapping techniques are applied to transform the similarity matrix into a visual representation; and finally, operations such as translation, rotation, and reflection are implemented to enhance the visual effects.

The web-based Java application, CiteSpace, is primarily designed to focus on the identification and investigation of cutting-edge advancements within a specific domain while examining their interconnections. Furthermore, CiteSpace facilitates the exploration of intrinsic links among diverse research domains by capturing keywords associated with highly influential citation bursts, thereby providing valuable insights into future research trends.

## 3. Results

### 3.1. Distribution of years

**Figure 2.**
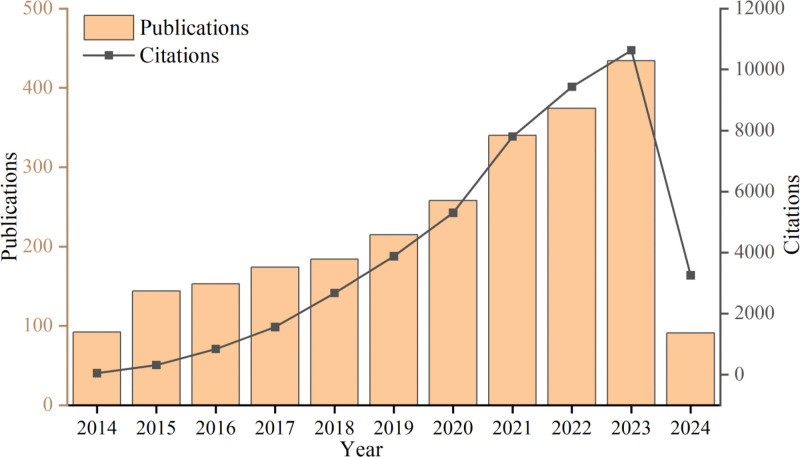
Number of publications and citations from 2014 to 2024.

The statistical findings depicted in Figure 2 illustrate a comprehensive quantitative analysis conducted on research papers exploring the implementation of ultrasound technology within the medical domain between 2014 and 2024. Notably, there has been a consistent annual growth observed in scholarly publications pertaining to ultrasound technology’s applications, substantiated by historical statistical trends. The analysis reveals a clear exponential growth pattern in the annual publication numbers, as evidenced by the fitting of a polynomial trendline (*y* = 20.218*x* + 102.24, *R*^2^ = 0.6046). The citation frequency of the literature has shown a consistent annual increase over the past decade in accordance with a linear trend (*y* = 901.68*x* − 1348.2, *R*^2^ = 0.6355). Nevertheless, it is crucial to acknowledge that this study’s temporal limitation extends until April 1^st^, 2024 and restricts research publications within the specified year.

### 3.2. Distribution of countries and institutions

The data presented in Table 2 reveals that China and the United States occupy the first and second positions, respectively, in terms of publication count, with 733 and 634 papers. The corresponding percentages, namely 29.81% and 25.78%, indicate a substantial level of interest from both nations in this specific field. The combined research output from these 2 countries accounts for 50% of all publications, highlighting their substantial involvement. Furthermore, based on the average citation index values, the United States (28.38), South Korea (27.96), and Australia (27.57) rank among the top 3, indicating significant advancements in research within this field and the attainment of relatively mature conclusions.

**Table 2:** Distribution of the top 10 countries and regions.

Rank	Country	Region	Quantity	Percentage/%	ACI	*h-index*
1	China	East Asia	733	29.81	15.03	47
2	USA	North America	634	25.78	28.38	60
3	Germany	Central Europe	167	6.79	27.29	33
4	England	Western Europe	161	6.55	25.65	33
5	Italy	South Europe	143	5.82	23.92	30
6	France	Western Europe	138	5.61	20.1	28
7	Canada	North America	135	5.49	26.48	28
8	India	South Asia	94	3.82	14.19	20
9	South Korea	East Asia	85	3.46	27.96	23
10	Australia	Australian	75	3.05	27.57	23

ACI = average citations per item.

The data presented in Table 3 illustrates that Harvard University has emerged as a prominent contributor in this field, with a total of 88 research publications. The Chinese Academy of Sciences diligently monitors the academic landscape through 82 publications, while the University of California System closely follows with 81 publications. The Mayo Clinic demonstrates an impressive average citations per item (ACI) value of 53.08, indicating significant research achievements within the scholarly community. The Pennsylvania Commonwealth System of Higher Education exhibits an ACI value of 47.81, while the University of Toronto’s ACI is marginally lower at 31.19, securing a third-place ranking.

**Table 3:** Distribution of the top 10 institutions.

Rank	Institution	Country	Quantity	SOTC	ACI
1	Harvard University	USA	88	1750	19.89
2	Chinese Academy of Sciences	China	82	1328	16.2
3	University of California System	California	81	1963	24.23
4	Centre National de la Recherche Scientifique Cnrs	France	68	1322	19.44
5	University of Texas System	USA	62	1093	17.63
6	University of London	England	56	1167	20.84
7	Mayo Clinic	USA	53	2813	53.08
8	University System of Ohio	USA	48	966	20.13
9	Pennsylvania Commonwealth System of Higher Education PCShe	USA	47	2247	47.81
10	University of Toronto	Canada	42	1310	31.19

ACI = average citations per item, PCShe = Pennsylvania Commonwealth System of Higher Education, SOTC = sum of the times cited

**Figure 3.**
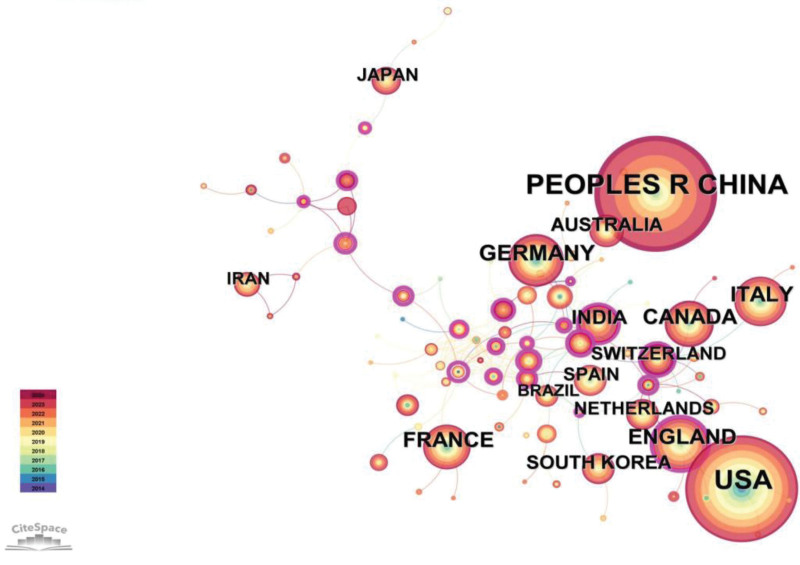
Co-occurrence map of countries.

**Figure 4.**
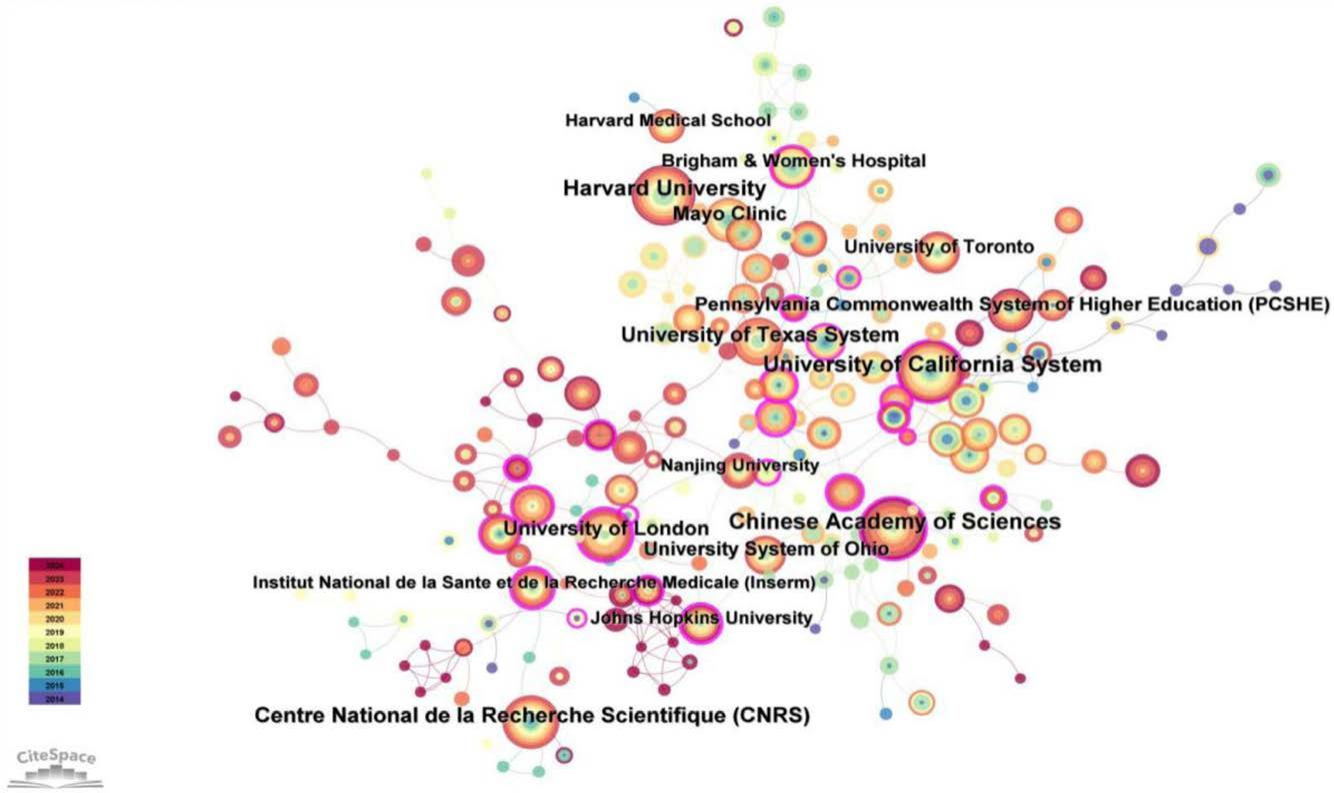
Co-occurrence map of institutions.

The data presented in Table 2 demonstrates that China and the United States occupy the first and second positions, respectively, in terms of publication count, with 733 and 634 papers. Throughout this period, a total of 90 countries and 3462 institutions actively participated in relevant research endeavors. The research capabilities of these nations have progressively strengthened, accompanied by enhanced collaboration among institutions, collectively contributing to the advancement of global scientific research. Figures 3 and 4 provide a more comprehensive portrayal of international collaboration in this field and effectively depict the collaborative relationships among various countries and institutions.

### 3.3. Distribution of author

Based on the findings presented in Table 4, Dietrich CF has emerged as the foremost contributor with an impressive total of 18 published articles, thus attaining the highest rank. It is worth noting that Glanc P from Canada and Cui XW from China have also made significant contributions by authoring an equal number of 11 publications each.

**Table 4:** Distribution of the top 10 authors.

Rank	Author	Country	Institute	TP	Total link strength
1	Dietrich CF	Germany	Caritas-krankenhaus Bad Mergentheim	18	26
2	Glanc P	Canada	Sunnybrook Hlth Sci Ctr	11	26
3	Cui XW	China	Zhengzhou Univ	11	20
4	Jiang XN	USA	N Carolina State Univ	11	3
5	Cash BD	USA	Univ S Alabama	9	21
6	Carucci LR	USA	Virginia Commonwealth Univ	8	22
7	Wang YY	China	Fudan Univ	8	9
8	Wang J	China	Zhejiang Prov Peoples Hosp	8	0
9	Sun HX	China	Jiangsu Univ	7	19
10	Moy L	USA	NYU	7	18

TP = total publications.

**Figure 5.**
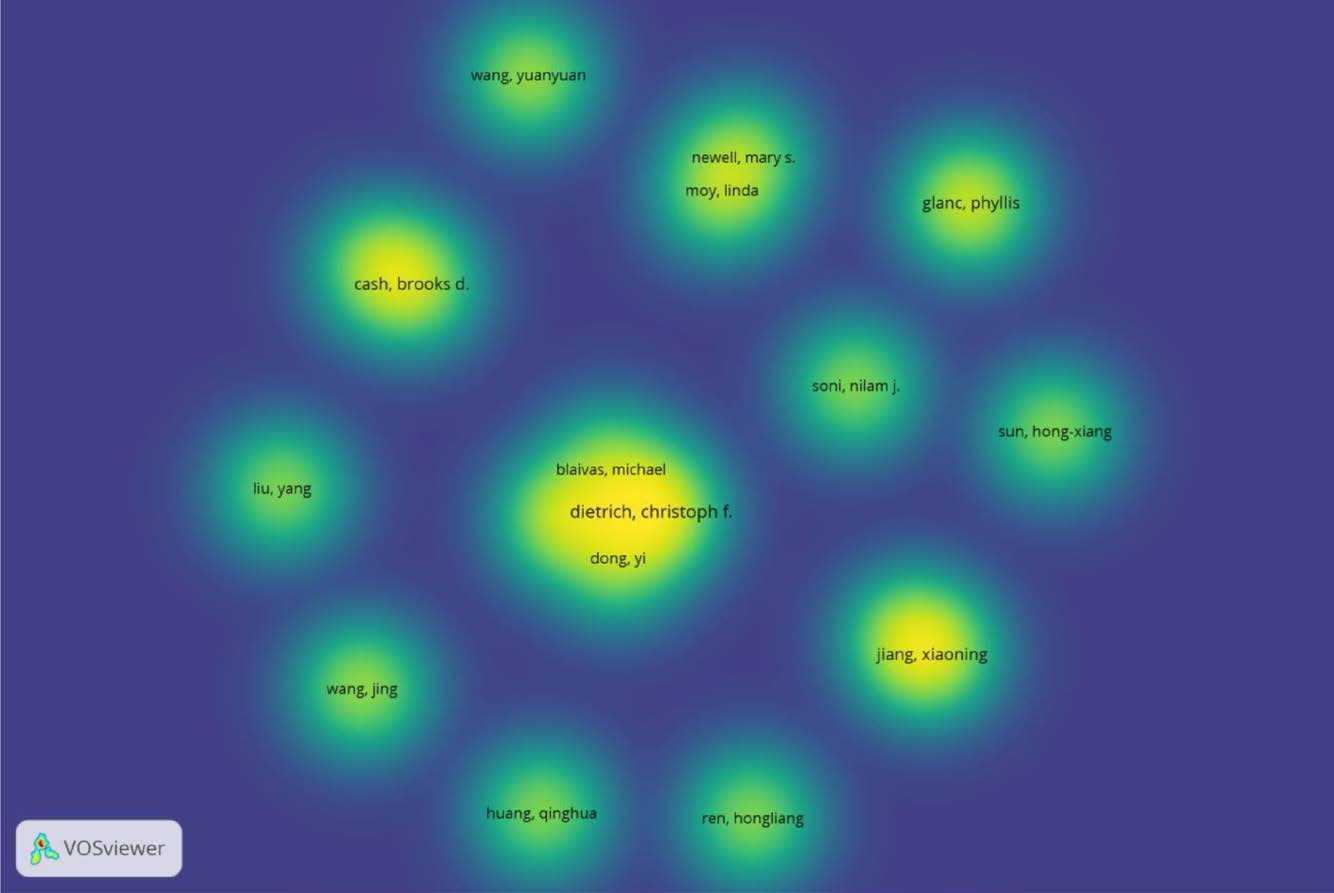
Cooperation map of authors in the studies of ultrasound’s medical application.

According to Price’s Law, it can be inferred that a minimum of 5 publications is necessary for an author to attain recognition as a core contributor within this specific discipline. The threshold for identifying core authors lies at or above 5 publications. By employing VOSviewer, the generation of a density visualization map showcasing prominent authors (as referenced in Fig. 5) becomes possible, enabling direct observation and unveiling the formation of a research community centered around Dietrich CF, Glanc P, Jiang XN, Cash BD, and Carucci LR.

### 3.4. Distribution of journal and WOS classification

According to Table 5, the analysis demonstrates that Radiology Nuclear Medicine Medical Imaging represents approximately18.42% of published papers, followed by Electrical Electronic Engineering at around 13.46% and Acoustics at about 10.70%. Moreover, an extensive range of significant disciplines are comprehensively addressed in the literature including Biomedical Engineering (approximately 10.53%), Applied Physics (around 7.56%), Multidisciplinary Materials Science (about 6.59%), Multidisciplinary Chemistry (approximately 6.43%), General Internal Medicine (roughly 5.94%), Instruments and Instrumentation (accounting for roughly 5.73%), as well as Nanoscience and Nanotechnology contributing to approximately 4.27%. These findings underscore both the broad spectrum encompassed by research fields within this domain and the employment of diverse methodologies.

**Table 5:** Distribution of the top 10 WOS categories.

Rank	Quantity	WOS categories	Percentage/%
1	453	Radiology Nuclear Medicine Medical Imaging	18.42
2	331	Engineering Electrical Electronic	13.46
3	263	Acoustics	10.70
4	259	Engineering Biomedical	10.53
5	186	Physics Applied	7.56
6	162	Materials Science Multidisciplinary	6.59
7	158	Chemistry Multidisciplinary	6.43
8	146	Medicine General Internal	5.94
9	141	Instruments Instrumentation	5.73
10	105	Nanoscience Nanotechnology	4.27

WOS = Web of Science.

The journal “IEEE Transactions on Ultrasonics Ferroelectronics and Frequency Control” demonstrates the highest publication count in this field, with a total of 87 articles, as evidenced in Table 6. Close contenders include “Journal of the American College of Radiology” (57) and “Sensors” (56). Notably among the journals ranked highly based on ACI values are “IEEE Transactions in Medical Imaging” (132.63), “IEEE Transactions in Biomedical Engineering” (37.08), and “Ultrasound in Medicine and Biology” (22.19).

**Table 6:** Distribution of the top 10 journals.

Rank	Journal title	Quantity	ACI
1	*IEEE Transactions on Ultrasonics Ferroelectrics and Frequency Control*	87	18.24
2	*Journal of the American College of Radiology*	57	15.98
3	*Sensors*	56	19.82
4	*Diagnostics*	35	7.77
5	*Applied Sciences Basel*	32	10.53
6	*Ultrasound in Medicine and Biology*	32	22.19
7	*Journal of Ultrasound in Medicine*	27	15.85
8	*IEEE Access*	26	12.88
9	*IEEE Transactions in Biomedical Engineering*	24	37.08
10	*IEEE Transactions in Medical Imaging*	24	132.63

ACI = average citations per item.

### 3.5. Highly cited literature analysis

In this section, as shown in Table 7, we present a comprehensive analysis of the top 15 most frequently cited articles to provide profound insights into the advancements and focal areas within this research domain. The article titled “Convolutional Neural Networks for Medical Image Analysis: Full Training or Fine Tuning?” has received the highest number of citations, focusing on the application of convolutional neural networks (CNNs) in medical image analysis with specific emphasis on their effectiveness in detecting subtle calcifications in digital mammography and lung nodules in CT datasets. CNNs possess inherent transferable knowledge, facilitating successful integration of pretrained CNNs’ embedded knowledge into the field of medical imaging.^[12]^

**Table 7:** Distribution of the top 15 cited documents.

Rank	Title	Type	Journal	Y	C	IN	CN
1	Convolutional neural networks for medical image analysis: full training or fine tuning?^[12]^	Article	*IEEE Transactions on Medical Imaging*	2016	1873	4	1
2	Holograms for acoustics^[13]^	Article	*Nature*	2016	597	2	1
3	Computer-aided diagnosis with deep learning architecture: applications to breast lesions in US images and pulmonary nodules in CT scans^[14]^	Article	*Scientific Reports*	2016	534	9	4
4	NiftyNet: a deep-learning platform for medical imaging^[18]^	Article	*Computer Methods and Programs in Biomedicine*	2018	351	5	1
5	Deep learning for classification and localization of COVID-19 markers in point-of-care lung ultrasound^[20]^	Article	*IEEE Transactions on Medical Imaging*	2020	302	8	2
6	On the opportunities and challenges in microwave medical sensing and imaging^[15]^	Article	*IEEE transactions on Biomedical Engineering*	2015	260	4	2
7	Ultrasound modulates ion channel currents^[23]^	Article	*Scientific Reports*	2016	229	4	1
8	Generalizing deep learning for medical image segmentation to unseen domains via deep stacked transformation^[24]^	Article	*IEEE Transactions on Medical Imaging*	2020	224	4	1
9	Imaging of sarcopenia: old evidence and new insights^[21]^	Article	*European Radiology*	2020	223	3	1
10	Programmable real-time clinical photoacoustic and ultrasound imaging system^[16]^	Article	*Scientific Reports*	2016	172	4	2
11	Organic semiconducting photoacoustic nanodroplets for laser-activatable ultrasound imaging and combinational cancer therapy^[24]^	Article	*ACS NANO*	2018	170	3	2
12	A mm-sized implantable medical device (IMD) with ultrasonic power transfer and a hybrid bi-directional data link^[25]^	Article	*IEEE Journal of Solid-State Circuits*	2015	166	1	1
13	Ultrasound in management of rheumatoid arthritis: ARCTIC randomized controlled strategy trial^[22]^	Article	*BMJ-British Medical Journal*	2016	156	13	2
14	Constrained deep weak supervision for histopathology image segmentation^[19]^	Article	*IEEE Transactions on Medical Imaging*	2017	150	3	1
15	Point of care ultrasound: a WFUMB position paper^[17]^	Article	*Ultrasound in Medicine and Biology*	2018	136	14	9

C = citations, CN = country number, IN = institute number, Y = year.

The article “Holograms for Acoustics” introduces the technology of acoustic holography, which enables precise manipulation of intricate sound fields in 3 dimensions, offering exceptional resolution and information content. This pioneering technique finds applications in medical imaging and innovative ultrasonic advancements.^[13]^

The article titled “Utilizing Deep Learning-Based Computer-Aided Diagnosis (CADx) for Discriminating Breast Cancer and Pulmonary Nodules” investigates the application of a deep learning architecture in computer-aided diagnosis to differentiate between breast lesions in US images and pulmonary nodules in CT scans. By employing a Stacked Denoising Auto-Encoder for both feature extraction and classification, this study effectively addresses potential inaccuracies inherent in conventional CADx algorithms.^[14]^

The remaining 12 papers showcase significant advancements in the field of medical imaging, with a multitude of studies providing indispensable support for disease diagnosis and treatment. Chandra’s research^[15]^ presents the principles, imaging techniques, and applications of microwave medical sensing and imaging, showcasing its potential in the field of medical diagnosis. The programmable real-time clinical photoacoustic and ultrasonic imaging system developed by Kim provides a robust tool for advanced medical research.^[16]^ The current status and future potential of instant ultrasound were deliberated by Dietrich, who underscored its pivotal role in clinical practice.^[17]^

The niftynet deep learning platform, developed by Gibson, provides a highly efficient solution for medical image analysis in the field of medical imaging^[18]^; Jia developed weakly supervised learning algorithm substantially improves the accuracy of histopathological image segmentation^[19]^; The implementation of deep learning technology by Roy facilitated the precise classification and localization of COVID-19 markers in lung ultrasound images, thereby introducing an innovative approach for disease diagnosis.^[20]^

The medical imaging diagnosis of sarcopenia was extensively discussed by Albano, encompassing a comprehensive analysis of various imaging modalities, measurement techniques, and diagnostic criteria^[21]^; Haavardsholm conducted a randomized controlled trial to compare the efficacy of different treatment strategies in the management of rheumatoid arthritis, thus providing valuable insights for clinical interventions.^[22]^

Kubanek has shown that focused ultrasound can modulate ion channel currents. This research has revealed its mechanism of action at the cellular level, thus laying a solid foundation for various multidisciplinary applications and the development of sonogenetics.^[23]^ Tang has presented the unique features of organic semiconductor photoacoustic nanodroplets, which are used for laser-activated ultrasound imaging and combined cancer therapy.^[24]^ Charthad has introduced millimeter-sized implantable medical devices with ultrasound power transmission and bidirectional data links, providing valuable assistance for in vivo medical applications.^[25]^

The future research agenda should prioritize the enhancement of accuracy and resolution in medical imaging technology, alongside further exploration of deep learning algorithms for precise disease diagnosis in medical image analysis. Simultaneously, the integration of multimodal medical imaging technology is anticipated to enable comprehensive analysis of diverse image information, thereby offering extensive support for early disease detection and treatment. Moreover, it is imperative to conduct additional research and discussions on safety and ethical concerns surrounding medical imaging technology to ensure its reliability and sustainability in clinical applications.

### 3.6. Clustering timeline analysis

**Figure 6.**
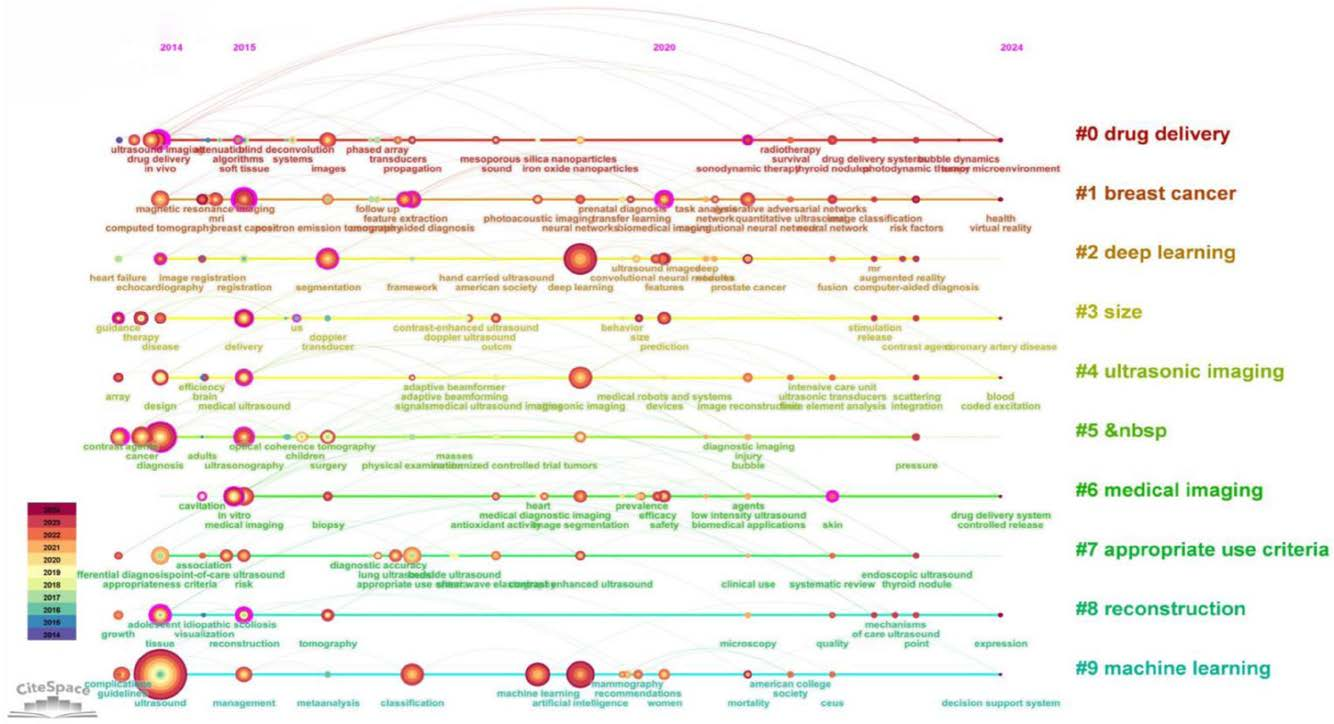
Timeline map of co-cited references.

As shown in Figure 6, the visual timeline map of the cluster was generated using CiteSpace, representing literature as nodes in cool and warm hues respectively, both prior to and following 2019. The data demonstrates that drug delivery, breast cancer, and deep learning have emerged as prominent research areas within this field in recent years.

### 3.7. Keywords analysis

The primary function of keywords is to encapsulate the fundamental content of an article and facilitate efficient identification of cutting-edge research within a specific knowledge domain. The data presented in Table 8 demonstrates that, apart from ultrasound (636), high-frequency terms include diagnosis (152), ultrasonography (132), deep learning (131), system (103), cancer (102), and classification (87). These keywords serve to unveil the focal points and trends within the current research domain.

**Table 8:** Distribution of the top 20 keywords.

Rank	Keywords	Occurrences	Total link strength	Rank	Keywords	Occurrences	Total link strength
1	Ultrasound	636	503	11	Machine Learning	75	184
2	Diagnosis	152	244	12	Management	74	97
3	Ultrasonography	132	145	13	Medical Imaging	72	94
4	Deep Learning	131	289	14	Imaging	70	104
5	System	103	146	15	Design	70	65
6	Cancer	102	164	16	Ultrasound Imaging	69	60
7	Classification	87	206	17	MRI	69	109
8	Artificial Intelligence	86	230	18	In vivo	65	59
9	Segmentation	82	144	19	Medical Ultrasound	65	46
10	Model	77	84	20	Ultrasonic Imaging	65	75

### 3.8. Co-occurrence and cluster analysis

**Figure 7.**
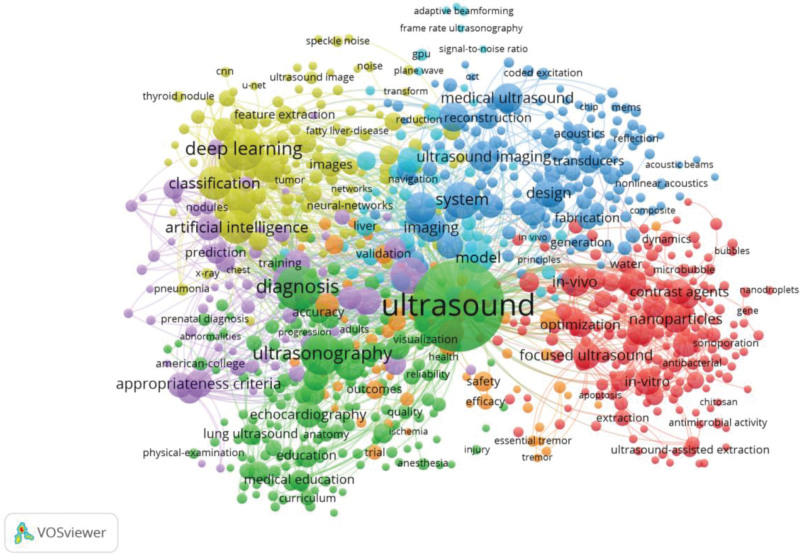
Co-occurrence map of keywords.

**Figure 8.**
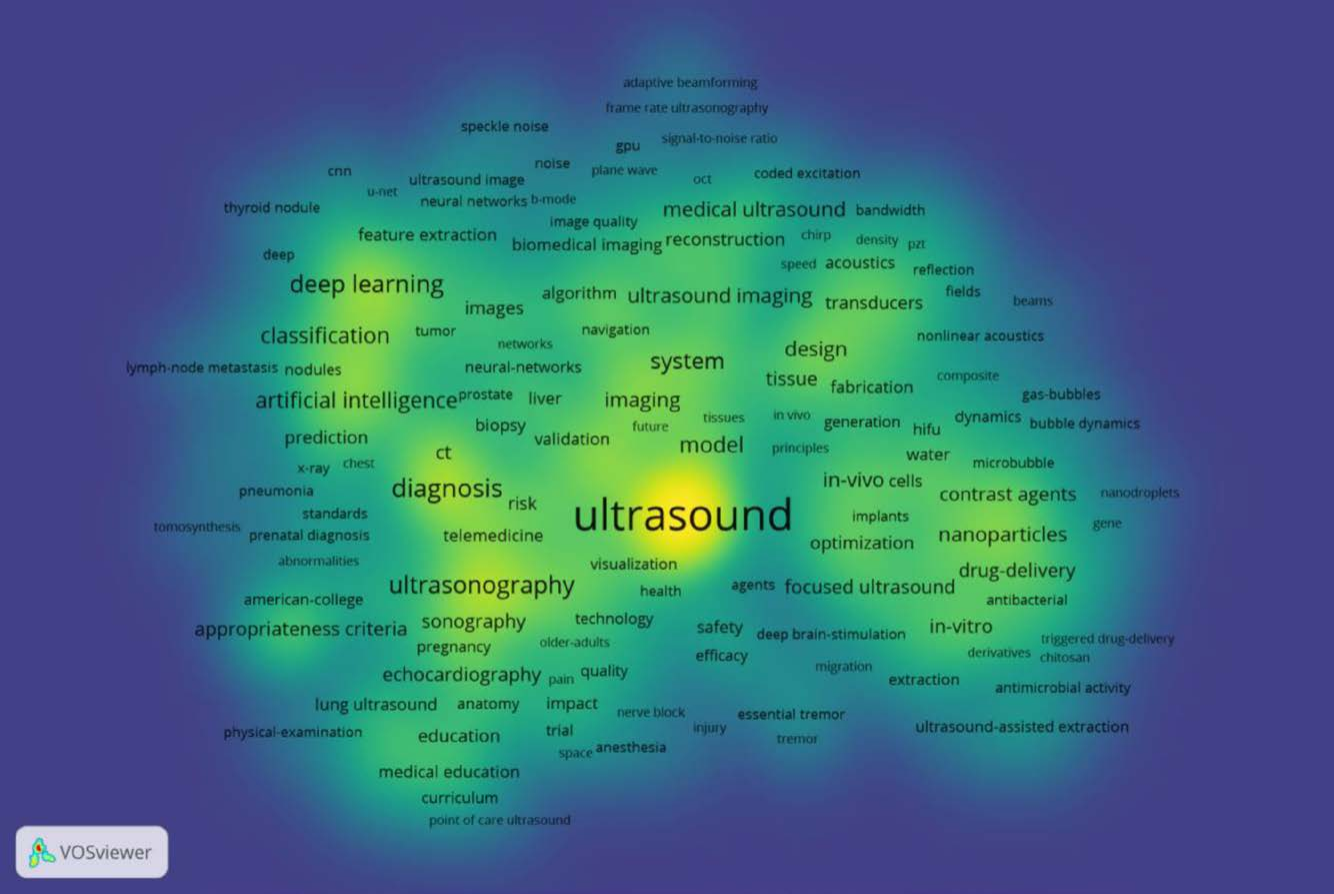
Density map of keywords.

The co-occurrence of keywords (Fig. 7) and the density diagram (Fig. 8) were visualized using VOSviewer. Cluster analysis was applied to identify 7 distinct clusters, where nodes sharing the same color indicate similar research themes. Among the top 3 clusters, the green cluster exhibits the highest node count, primarily focusing on 2 major themes: “Diagnosis” and “Ultrasonography,” thereby emphasizing the pivotal role of ultrasound technology in disease diagnosis. The terms “Ultrasound,” “Diagnosis,” “Ultrasonography,” and “Deep Learning” demonstrate significant prominence, as evidenced by the keyword density diagram.

### 3.9. Emergence analysis

The detection of abrupt changes in keyword frequency signifies significant fluctuations in the occurrence of keywords within short time intervals, thereby facilitating the identification of emerging trends in a specific research field and subsequently enabling the prediction of future research directions.^[26]^ The top 15 keywords demonstrating sudden increases in frequency are presented in Table 9. Ultrasound contrast agents, artificial intelligence (AI), machine learning, prediction, and stimulation have emerged as the prevailing research themes during recent years (2020–2024).

**Table 9:** Top 15 keywords with the strongest citation bursts.

Keywords	Year	Strength	Begin	End	2014–2024
In vivo	2014	7.38	2014	2017	▂▃▃▃▃▂▂▂▂▂▂▂
Surgery	2014	5.12	2014	2018	▂▃▃▃▃▃▂▂▂▂▂▂
Clinical application	2014	4.56	2014	2016	▂▃▃▃▂▂▂▂▂▂▂▂
Acoustic radiation force	2014	4.07	2014	2018	▂▃▃▃▃▃▂▂▂▂▂▂
Radiation force	2014	3.8	2014	2015	▂▃▃▂▂▂▂▂▂▂▂▂
Elasticity	2015	5.61	2015	2018	▂▂▃▃▃▃▂▂▂▂▂▂
Ultrasound elastography	2015	4.41	2016	2017	▂▂▂▃▃▂▂▂▂▂▂▂
Acoustic droplet vaporization	2014	4.14	2018	2019	▂▂▂▂▂▃▃▂▂▂▂▂
Antioxidant activity	2017	4.39	2020	2024	▂▂▂▂▂▂▂▃▃▃▃▃
Diagnostic accuracy	2018	4.07	2020	2022	▂▂▂▂▂▂▂▃▃▃▂▂
Ultrasound contrast agents	2018	3.66	2020	2021	▂▂▂▂▂▂▂▃▃▂▂▂
Artificial intelligence	2019	7.11	2021	2024	▂▂▂▂▂▂▂▂▃▃▃▃
Machine learning	2019	5.01	2021	2024	▂▂▂▂▂▂▂▂▃▃▃▃
Prediction	2021	4.53	2021	2024	▂▂▂▂▂▂▂▂▃▃▃▃
Stimulation	2021	3.68	2021	2024	▂▂▂▂▂▂▂▂▃▃▃▃

## 4. Discussion

### 4.1. Research status

A comprehensive bibliometric analysis was conducted using information visualization tools such as CiteSpace and VOSviewer to investigate scholarly publications pertaining to ultrasound technology in the medical field from 2014 to 2024. This meticulous examination encompassed author contributions, significant literature pieces, emerging research patterns, and innovative advancements. By conducting a thorough word-by-word assessment, we were able to identify prominent areas of study during each time period while elucidating the underlying trajectory of topic evolution. Through this rigorous investigative process, we successfully identified current cutting-edge research frontiers in ultrasound technology within medicine that will serve as a foundation for future discussions.

The period from 2014 to 2024 witnessed a remarkable exponential growth trend in research on ultrasound technology within the medical field, indicating a continuous expansion in both the depth and impact of such research. This phenomenon serves as strong evidence for the prevalence and extensive application of ultrasound technology in the medical field. The investigation of this subject saw active participation from a total of 12,196 authors, resulting in the publication of 2459 scholarly works, as indicated by statistical data. The study encompassed a total of 3462 institutions spanning across 90 countries. Currently, research in this field primarily focuses on experimental investigations, clinical studies, and comprehensive literature reviews. The frequency of inter-institutional cooperation has been increasing steadily, thereby facilitating advancements in this domain. The distribution of journals reflects the credibility and impact of research findings related to medical applications for ultrasound technology.

The data released by the journal reveals that China and the United States demonstrate a predominant presence in terms of publication volume. The research output from these 2 countries exceeds 50%, underscoring their exceptional scientific capabilities and substantial investments in relevant fields. Although the publication output from South Korea and Australia may be relatively lower compared to that of China and the United States, their exceptional ACI values underscore their remarkable research quality. The field of ultrasound technology has witnessed substantial advancements in Europe and America, leading to the acquisition of profound knowledge and remarkable achievements. Substantial progress has been made in ultrasound technology in Europe and America, leading to a wealth of knowledge and accomplishments.^[27]^

The data presented in Table 2 indicates that China and the United States occupy the first and second positions, respectively, in terms of publication count, with 733 and 634 papers. Leading institutions such as Harvard University, the Chinese Academy of Sciences, and the University of California System are spearheading cutting-edge research in ultrasound technology. The esteemed research team at Harvard University is dedicated to advancing drug delivery through comprehensive investigations, with the aim of optimizing the precision and control of pharmaceutical release within the human body.^[28]^ The research carried out at the Chinese Academy of Sciences primarily concentrates on harnessing cutting-edge ultrasound technology to improve disease characterization when diagnosing breast lesions through ultrasound imaging.^[29]^ The primary objective of the research conducted at the University of California System is to advance photoacoustic imaging systems with a focus on enhancing imaging resolution.^[30]^ Revered for his prolific publication record, Dietrich CF has dedicated himself to conducting extensive research on point-of-care ultrasound.^[17]^ Upon further investigation, it has been discovered that 11 publications demonstrate collaborative efforts involving 3 or more institutions, while 8 publications exemplify international cooperation spanning across at least 2 countries. The research conducted within this domain is significantly influenced by interdisciplinary and global collaborations, which have a profound impact on its advancement. Collaborative interactions among prestigious institutions and nations make substantial contributions to the rapid progress of this field, resulting in more influential research findings. These partnerships among authors, organizations, and nations play an indispensable role in fostering innovation and propelling advancements within the medical field.

The classification data of articles and the distribution data of journals reveal a salient characteristic in ultrasonic applications within the field of medical research. This distinctive attribute fosters innovative advancements across fundamental and applied disciplines, as well as interdisciplinary collaborations, thereby making a substantial contribution to the enhancement of human health. The inclusion of this feature facilitates the promotion of innovative development in the medical field and significantly contributes to improvement. Based on statistical data, it can be inferred that despite having a lower publication count compared to top-tier journals, “IEEE Transactions in Medical Imaging” demonstrates a remarkably high ACI value, indicating the substantial influence exerted by its articles.

### 4.2. Hotspot analysis and prediction

#### 4.2.1. AI and deep learning in ultrasonic technology

The integration of AI and deep learning into ultrasound technology has become increasingly indispensable in the field of medical diagnosis. Firstly, NiftyNet is a specialized deep learning platform specifically tailored for medical imaging applications, aiming to alleviate the barriers to entry for AI technology in this domain. It provides comprehensive support for diverse types of medical image analysis and employs a modular architecture, thereby facilitating the engagement of healthcare professionals in deep learning research on medical images.^[18]^ Additionally, a research team has proposed a deep learning-based approach for the analysis of lung ultrasound images. This method has demonstrated remarkable efficacy in assisting medical professionals with identifying imaging patterns associated with COVID-19, thereby enhancing diagnostic accuracy and elucidating imaging biomarkers for this disease.^[20]^ The significance of this cannot be overstated in the battle against the COVID-19 pandemic, as it equips clinicians with a powerful diagnostic tool. In contrast, Romeo study conducted a retrospective analysis of 201 breast lesions in 174 patients, aiming to compare the diagnostic accuracy between AI and sonographers. The findings revealed that AI achieved a diagnostic accuracy rate of 82% in detecting breast lesions, which is comparable to the diagnostic accuracy rate of sonographers (79.4%).^[31]^ The statement suggests that AI demonstrates significant practicality in the field of breast lesion diagnosis. The incorporation of AI and deep learning techniques into ultrasound technology has led to notable advancements in the medical domain, thereby highlighting the immense potential of deep learning technology.

#### 4.2.2. Ultrasound stimulation and ablation technology

Currently, significant advancements are being made in both the research and application of ultrasound stimulation and ablation technology within the biomedical field. Through an extensive investigation into its impact on potassium ion channel currents across cell membranes, researchers have unveiled the modulatory potential of ultrasound. Moreover, it has been observed that this modulation is intricately linked to both the intensity of ultrasound and the specific type of channel.^[23]^ The discovery presents a novel theoretical framework for the application of ultrasound in the field of biomedicine. The study focused on patients diagnosed with PD, where researchers specifically selected individuals exhibiting tremor-type PD as subjects for dual-target stimulation of the Vim and globus pallidus thalamic bundles. The experimental findings demonstrated that patients exhibited significant improvements in tremor, rigidity, and postural abnormalities following ultrasound stimulation treatment.^[32]^ The findings suggest that ultrasound stimulation exhibits therapeutic efficacy in ameliorating symptoms associated with PD. Additionally, a research team has identified that low-intensity focused ultrasound can induce dopamine release in PC12 cells and PD model mice.^[33]^ The authors propose that this phenomenon is associated with the enhancement of neuronal regeneration and improvement of membrane permeability through ultrasound, thereby providing a novel avenue for investigating the application of ultrasound in the field of neural regeneration. High-intensity focused ultrasound ablation technology finds extensive clinical applications primarily for ablating and treating conditions such as uterine fibroids,^[34]^ prostate cancer,^[35]^ pancreatic cancer,^[36]^ renal tumors,^[37]^ and breast tumors.^[38]^ Focused ultrasound ablation enables precise eradication of tumor tissues while minimizing collateral damage to surrounding normal tissues, thereby effectively achieving therapeutic objectives. Overall, ultrasound stimulation and ablation technology exhibit substantial potential for biomedical applications. Moreover, ultrasound technology plays a pivotal role in modulating potassium ion channel currents, ameliorating symptoms associated with PD, and treating tumors.

#### 4.2.3. Ultrasound-assisted extraction

The application of ultrasound-assisted extraction technology has garnered significant attention in recent years, particularly showcasing remarkable efficacy in the extraction and analysis of proteins within intricate mixtures. Extensive research findings have unveiled the efficient protein extraction capability of ultrasound-assisted extraction from corn, which holds substantial implications for the food and feed industries.^[39]^ Additionally, extensive research has been conducted on the application of ultrasound-assisted extraction in the field of plant extracts. Taking *Erica carnea* L as a case study, extracts obtained through both ultrasound-assisted and conventional extraction methods demonstrated remarkable antioxidant, cytotoxic, and antibacterial activities. The active components exhibit tremendous potential applications in the fields of medicine and health products. The extraction of active components yields significantly divergent outcomes depending on the employed methodology. Ultrasound-assisted extraction has been observed to surpass non-ultrasound-assisted extraction in terms of efficacy.^[40]^ Expanding upon the ultrasound-assisted extraction technology, researchers have devised a novel β-cyclodextrin-enhanced extraction method that significantly amplifies the efficacy and stability of red beet extract. This innovative approach presents a promising solution for optimizing agricultural production.^[41]^ The application of ultrasound-assisted extraction technology offers significant advantages in the extraction and analysis of proteins and active components within complex mixtures. With continuous advancements in scientific research and technological innovations, ultrasound-assisted extraction is expected to find broader applications across various industries including food, pharmaceuticals, and others, thereby making a substantial contribution to human health and well-being.

#### 4.2.4. Ultrasonic drug delivery

The analysis of emerging terms suggests that ultrasonic drug delivery is poised to emerge as a predominant research direction in the future. The remote and noninvasive nature of ultrasound technology enables precise stimulation of nanocarrier release, thereby regulating drug distribution and enhancing targeted delivery within the body.^[42]^ Moreover, in the management of neurodegenerative disorders, ultrasound represents a secure and nonintrusive method that can selectively disrupt the blood–brain barrier. Consequently, this offers an innovative approach for delivering macromolecular drugs systemically to the brain.^[43]^

### 4.3. Advantages and limitations

The present study employs bibliometrics for the first time to systematically investigate the focal areas and cutting-edge issues in the application domain of ultrasound technology in medicine, thereby enhancing the methodological rigor and precision of this research endeavor through the utilization of 2 software programs for bibliometric analysis. Nevertheless, certain limitations persist in this study. Despite the utilization of the globally recognized and comprehensive journal citation index database WOS, conducting bibliometric analysis solely on it may inadvertently overlook other relevant literature sources. Furthermore, the inclusion of exclusively English-language literature could potentially introduce biases in terms of literature selection.

## 5. Conclusion

In the past decade, a comprehensive examination has been conducted to assess the current status of ultrasound technology in medical applications. This analysis has been facilitated by employing suitable software that enables an intuitive and visually appealing representation of key areas under investigation. By extensively exploring emerging trends in development and cutting-edge research domains, it becomes evident that AI, deep learning techniques, guidance systems, and ultrasound-assisted extraction methods have emerged as central focal points. Moreover, both ultrasound stimulation techniques and drug delivery mechanisms exhibit significant potential as prominent avenues for further exploration in future studies. Consequently, this study serves as a valuable resource for selecting specific research directions within the field of ultrasound medicine.

## Author contributions

**Data curation:** Zhao Chenhao.

**Formal analysis:** Zhao Chenhao.

**Writing – original draft:** Zhou Ruqi, Sun Xiaozhuo.

**Writing – review & editing:** Wang Jiabo, Liu Jiao, Mu Yajuan.

## References

[1] Gu JJ Jing Y. Modeling of wave propagation for medical ultrasound: a review. IEEE Trans Ultrason Ferroelectr Freq Control. 2015;62:1979–93. 26559627 10.1109/TUFFC.2015.007034

[2] Fujii H Li SH Wu J. Repeated and targeted transfer of angiogenic plasmids into the infarcted rat heart via ultrasound targeted microbubble destruction enhances cardiac repair. Eur Heart J. 2011;32:2075–84. 21196445 10.1093/eurheartj/ehq475

[3] Wu MY Chen WT Chen Y. Focused ultrasound-augmented delivery of biodegradable multifunctional nanoplatforms for imaging-guided brain tumor treatment. Adv Sci. 2018;5:1700474. 10.1002/advs.201700474 PMC5908350 29721406

[4] Tufail Y Matyushov A Baldwin N. Transcranial pulsed ultrasound stimulates intact brain circuits. Neuron. 2010;66:681–94. 20547127 10.1016/j.neuron.2010.05.008

[5] Zhou H Niu LL Xia XX. Wearable ultrasound improves motor function in an MPTP mouse model of Parkinson’s disease. IEEE Trans Biomed Eng. 2019;66:3006–13. 30794160 10.1109/TBME.2019.2899631

[6] Zhang DQ Li HD Sun JF. Antidepressant-like effect of low-intensity transcranial ultrasound stimulation. IEEE Trans Biomed Eng. 2019;66:411–20. 29993461 10.1109/TBME.2018.2845689

[7] Hu YX Yang R Liu SM Wang H. Bibliometric analysis of transforaminal lumbar interbody fusion in lumbar spine surgery. Eur Rev Med Pharmacol Sci. 2024;28:907–23. 38375731 10.26355/eurrev_202402_35328

[8] Wang Y Sun Y Hu Y Xiao Z. Bibliometric analysis of anesthetic drugs’ effects on immune function- current knowledge. hotspots and future perspectives. Drug Des Devel Ther. 2023;17:3219–30. 10.2147/DDDT.S433629 PMC10615110 37908313

[9] Hu Y Yang R Liu S Wang H. Bibliometric analysis of interspinous device in treatment of lumbar degenerative diseases. Medicine (Baltim). 2024;103:e37351. 10.1097/MD.0000000000037351 PMC10906630 38428868

[10] Van Eck NJ Waltman L. Software survey: VOSviewer, a computer program for bibliometric mapping. Scientometrics. 2010;84:523–38. 20585380 10.1007/s11192-009-0146-3 PMC2883932

[11] Chen C. Science mapping: a systematic review of the literature. J Data Inform Sci. 2017;2:1–40.

[12] Tajbakhsh N Shin JY Gurudu SR Hurst RT Kendall CB Gotway MB Jianming L. Convolutional neural networks for medical image analysis: full training or fine tuning? IEEE Trans Med Imaging. 2016;35:1299–312. 26978662 10.1109/TMI.2016.2535302

[13] Melde K Mark AG Qiu T Fischer P. Holograms for acoustics. Nature. 2016;537:518–22. 27652563 10.1038/nature19755

[14] Cheng JZ Ni D Chou YH. Computer-aided diagnosis with deep learning architecture: applications to breast lesions in US images and pulmonary nodules in CT scans. Sci Rep. 2016;6:24454. 27079888 10.1038/srep24454 PMC4832199

[15] Chandra R Zhou H Balasingham I Narayanan RM. On the opportunities and challenges in microwave medical sensing and imaging. IEEE Trans Biomed Eng. 2015;62:1667–82. 25993698 10.1109/TBME.2015.2432137

[16] Kim J Park S Jung Y. Programmable real-time clinical photoacoustic and ultrasound imaging system. Sci Rep. 2016;6:35137. 27731357 10.1038/srep35137 PMC5059665

[17] Dietrich CF Goudie A Chiorean L. Point of care ultrasound: a WFUMB position paper. Ultrasound Med Biol. 2017;43:49–58. 27472989 10.1016/j.ultrasmedbio.2016.06.021

[18] Gibson E Li W Sudre C. NiftyNet: a deep-learning platform for medical imaging. Comput Methods Programs Biomed. 2018;158:113–22. 29544777 10.1016/j.cmpb.2018.01.025 PMC5869052

[19] Jia Z Huang X Chang EI Xu Y. Constrained deep weak supervision for histopathology image segmentation. IEEE Trans Med Imaging. 2017;36:2376–88. 28692971 10.1109/TMI.2017.2724070

[20] Roy S Menapace W Oei S. Deep learning for classification and localization of COVID-19 markers in point-of-care lung ultrasound. IEEE Trans Med Imaging. 2020;39:2676–87. 32406829 10.1109/TMI.2020.2994459

[21] Albano D Messina C Vitale J Sconfienza LM. Imaging of sarcopenia: old evidence and new insights. Eur Radiol. 2020;30:2199–208. 31834509 10.1007/s00330-019-06573-2

[22] Haavardsholm EA Aga AB Olsen IC. Ultrasound in management of rheumatoid arthritis: ARCTIC randomised controlled strategy trial. BMJ. 2016;354:i4205. 27530741 10.1136/bmj.i4205 PMC4986519

[23] Kubanek J Shi J Marsh J Chen D Deng C Cui J. Ultrasound modulates ion channel currents. Sci Rep. 2016;6:24170. 27112990 10.1038/srep24170 PMC4845013

[24] Tang W Yang Z Wang S. Organic semiconducting photoacoustic nanodroplets for laser-activatable ultrasound imaging and combinational cancer therapy. ACS Nano. 2018;12:2610–22. 29451774 10.1021/acsnano.7b08628

[25] Charthad J Weber MJ Chang TC Arbabian A. A mm-sized implantable medical device (IMD) with ultrasonic power transfer and a hybrid bi-directional data link. solid-state circuits. IEEE J Solid-State Circuits. 2015;50:1741–53.

[26] Lin WW Luo YY Liu F. Status and trends of the association between diabetic nephropathy and diabetic retinopathy from 2000 to 2021: bibliometric and visual analysis. Front Pharmacol. 2022;13:937759. 35795563 10.3389/fphar.2022.937759 PMC9251414

[27] Daly SM Leahy MJ. “Go with the flow ‘: a review of methods and advancements in blood flow imaging. J Biophotonics. 2013;6:217–55. 22711377 10.1002/jbio.201200071

[28] Wang JL Kaplan JA Colson YL. Mechanoresponsive materials for drug delivery: Harnessing forces for controlled release. Adv Drug Deliv Rev. 2017;108:68–82. 27856307 10.1016/j.addr.2016.11.001 PMC5285479

[29] Xue C Zhu L Fu HZ. Global guidance network for breast lesion segmentation in ultrasound images. Med Image Anal. 2021;70:101989. 33640719 10.1016/j.media.2021.101989

[30] Hariri A Lemaster J Wang JX. The characterization of an economic and portable LED-based photoacoustic imaging system to facilitate molecular imaging. Photoacoustics. 2018;9:10–20. 29234601 10.1016/j.pacs.2017.11.001 PMC5723278

[31] Romeo V Cuocolo R Apolito R. Clinical value of radiomics and machine learning in breast ultrasound: a multicenter study for differential diagnosis of benign and malignant lesions. Eur Radiol. 2021;31:9511–9. 34018057 10.1007/s00330-021-08009-2 PMC8589755

[32] Chen JC Lu MK Chen CM Tsai C-H. Stepwise dual-target magnetic resonance-guided focused ultrasound in tremor-dominant Parkinson disease: a feasibility study. World Neurosurg. 2023;171:e464–70. 36563853 10.1016/j.wneu.2022.12.049

[33] Xu T Lu XX Peng DH. Ultrasonic stimulation of the brain to enhance the release of dopamine – a potential novel treatment for Parkinsons disease. Ultrason Sonochem. 2020;63:104955. 31945561 10.1016/j.ultsonch.2019.104955

[34] Pu YZK Jin C. Effectiveness and safety of high-intensity focused ultrasound in treatment of hepatocellular carcinoma adjacent to large vessels. Tumor. 2017;37:497–503.

[35] Sundaram KM Chang SS Penson DF Arora S. Therapeutic ultrasound and prostate cancer. Seminars Interv Radiol. 2017;34:187–200. 10.1055/s-0037-1602710 PMC5453783 28579687

[36] Marinova M Rauch M Mücke M. High-intensity focused ultrasound (HIFU) for pancreatic carcinoma: evaluation of feasibility, reduction of tumour volume and pain intensity. Eur Radiol. 2016;26:4047–56. 26886904 10.1007/s00330-016-4239-0

[37] Klatte T Marberger M. High-intensity focused ultrasound for the treatment of renal masses: current status and future potential. Curr Opin Urol. 2009;19:188–91. 19188773 10.1097/MOU.0b013e328323f641

[38] Zhao WP Han ZY Zhang J Liang P. A retrospective comparison of microwave ablation and high intensity focused ultrasound for treating symptomatic uterine fibroids. Eur J Radiol. 2015;84:413–7. 25572326 10.1016/j.ejrad.2014.11.041

[39] Darie-Ion L Jayathirtha M Hitruc GE. A proteomic approach to identify zein proteins upon eco-friendly ultrasound-based extraction. Biomolecules. 2021;11:1838. 34944482 10.3390/biom11121838 PMC8699583

[40] Velickovic V Durovic S Radojkovic M. Application of conventional and non-conventional extraction approaches for extraction of *Erica carnea* L.: Chemical profile and biological activity of obtained extracts. J Supercrit Fluids. 2017;128:331–7.

[41] Tutunchi P Roufegarinejad L Hamishehkar H Alizadeh A. Extraction of red beet extract with β-cyclodextrin-enhanced ultrasound assisted extraction: a strategy for enhancing the extraction efficacy of bioactive compounds and their stability in food models. Food Chem. 2019;297:124994. 31253277 10.1016/j.foodchem.2019.124994

[42] Xia HS Zhao Y Tong R. Ultrasound-mediated polymeric micelle drug delivery [M]//ESCOFFRE J M, BOUAKAZ A. Therapeutic Ultrasound. 2016:365–84. 10.1007/978-3-319-22536-4_20 26486348

[43] Chen H Chen CC Acosta C Wu S-Y Sun T Konofagou EE. A new brain drug delivery strategy: focused ultrasound-enhanced intranasal drug delivery. PLoS One. 2014;9:e108880. 25279463 10.1371/journal.pone.0108880 PMC4184840

